# The Two-Way Relationship Between Calcium and Metabolism in Cancer

**DOI:** 10.3389/fcell.2020.573747

**Published:** 2020-11-13

**Authors:** Camille Dejos, Dimitra Gkika, Anna Rita Cantelmo

**Affiliations:** ^1^Univ. Lille, Inserm, U1003 - PHYCEL - Physiologie Cellulaire, Lille, France; ^2^Univ. Lille, CNRS, INSERM, CHU Lille, Centre Oscar Lambret, UMR 9020-UMR 1277-Canther-Cancer Heterogeneity, Plasticity and Resistance to Therapies, Lille, France; ^3^Institut Universitaire de France (IUF), Paris, France

**Keywords:** calcium, signaling, metabolism, interplay, cancer

## Abstract

Calcium ion (Ca^2+^) signaling is critical to many physiological processes, and its kinetics and subcellular localization are tightly regulated in all cell types. All Ca^2+^ flux perturbations impact cell function and may contribute to various diseases, including cancer. Several modulators of Ca^2+^ signaling are attractive pharmacological targets due to their accessibility at the plasma membrane. Despite this, the number of specific inhibitors is still limited, and to date there are no anticancer drugs in the clinic that target Ca^2+^ signaling. Ca^2+^ dynamics are impacted, in part, by modifications of cellular metabolic pathways. Conversely, it is well established that Ca^2+^ regulates cellular bioenergetics by allosterically activating key metabolic enzymes and metabolite shuttles or indirectly by modulating signaling cascades. A coordinated interplay between Ca^2+^ and metabolism is essential in maintaining cellular homeostasis. In this review, we provide a snapshot of the reciprocal interaction between Ca^2+^ and metabolism and discuss the potential consequences of this interplay in cancer cells. We highlight the contribution of Ca^2+^ to the metabolic reprogramming observed in cancer. We also describe how the metabolic adaptation of cancer cells influences this crosstalk to regulate protumorigenic signaling pathways. We suggest that the dual targeting of these processes might provide unprecedented opportunities for anticancer strategies. Interestingly, promising evidence for the synergistic effects of antimetabolites and Ca^2+^-modulating agents is emerging.

## Introduction

Calcium ions (Ca^2+^) are second messengers involved in signaling in many physiological processes, including the regulation of metabolic pathways (Clapham, 2007). Collectively termed the “Ca^2+^ transportome,” a variety of Ca^2+^ channels and transporters ensure Ca^2+^ flux between the extracellular space and the cytosol and between organelles within the cell, such as the endoplasmic reticulum (ER) and mitochondria, which act as major Ca^2+^ storage sites (Cardenas et al., 2018). Once Ca^2+^ enters the cell, some of the intracellular Ca^2+^ moves into the ER through energy-dependent sarco/endoplasmic reticulum calcium ATPases (SERCAs). However, following stimulation by extracellular stimuli and intracellular signals, the activation of two receptors, the inositol 1,4,5-triphosphate receptor (IP3R) and ryanodine receptor (RYR), mobilizes Ca^2+^ from this store, creating cytosolic Ca^2+^ hotspots in close proximity to mitochondria (Clapham, 2007). Mitochondrial Ca^2+^ uptake via voltage-dependent anion-selective channel protein (VDAC) and mitochondrial calcium uniporter protein (MCU) is a major regulator of several mitochondrial processes, including energy production and cell death (Duchen, 2000; Finkel et al., 2015). An increase in cytosolic Ca^2+^ levels also activates several Ca^2+^-binding proteins that are major players in signaling pathways and are able to directly regulate a variety of enzymes, transportome function, and gene expression (Berridge et al., 2000).

In recent years, a growing number of studies have yielded significant insights into how Ca^2+^ integrates with multiple metabolic pathways within cells (Rossi et al., 2019). This interaction is primarily related to the ability of Ca^2+^ to allosterically regulate the activity of key metabolic enzymes, such as mitochondrial dehydrogenases, thus impacting biosynthetic and energy-generating pathways. Furthermore, Ca^2+^ signaling can influence the generation of reactive oxygen species (ROS) at different subcellular locations. In turn, many Ca^2+^ channels and transporters are regulated by redox modifications.

Most of our knowledge of the integration of Ca^2+^ signaling with metabolic processes has come from the cardiovascular system and organ physiology, with well-known examples of Ca^2+^ coupling workload and energy metabolism in cardiomyocytes and skeletal muscle. Spikes in intracellular Ca^2+^ levels during contractile activity lead to changes in glucose metabolism by enhancing glucose uptake and transport (Wright et al., 2004; Waller et al., 2015). In insulin-sensitive tissues, the posttranslational activation of glucokinase (one of the rate-limiting enzymes of glycolysis) requires an increase in cytoplasmic Ca^2+^ levels (Markwardt et al., 2016). Variations in Ca^2+^ oscillations also regulate the generation of glucose from non-carbohydrate precursors (gluconeogenesis) (Gaspers et al., 2019) by modulating the expression of genes related to glucose metabolism in the liver (Wang et al., 2012; Zou et al., 2018). Furthermore, in response to Ca^2+^-mobilizing hormones, Ca^2+^ controls the phosphorylation state, and hence the activity, of key enzymes regulating glucose storage (glycogen) (Amaya and Nathanson, 2013). The relevance of these observations is underscored by studies showing an increase in intracellular Ca^2+^ levels and Ca^2+^-dependent signaling in obese and diabetic mice (Ozcan et al., 2012; Wang et al., 2012). In these pathological conditions, high glucose concentrations increase cytosolic Ca^2+^ levels by stimulating extracellular Ca^2+^ influx, ultimately leading to cytosolic Ca^2+^ overload and cellular damage (Song et al., 2003).

Nevertheless, it is difficult to clearly separate the effects of Ca^2+^ from the metabolic changes associated with muscle contraction or insulin sensitivity, and the interpretation of this interaction is often hindered by the causality dilemma. Although the development of sensitive methods has allowed for the recording of subtle, transient and localized intracellular Ca^2+^ peaks in response to metabolic changes, much remains to be understood regarding the reciprocal regulatory role of metabolism.

The “chicken and egg” situation is particularly evident in cancer cells, where both Ca^2+^ signaling and metabolism are aberrant, and it is therefore difficult to define whether dysregulated Ca^2+^ homeostasis leads to metabolic reprogramming or vice versa. Moreover, since much of the research investigating Ca^2+^ signaling in cancer has focused on evaluating relevant functional processes (such as proliferation, migration, and survival) as a result of aberrant expression levels of channels, pumps and transporters, the contribution of this interplay to cancer pathogenesis has often been overlooked.

One of the strategies adopted by cancer cells to sustain their high proliferation rate and survival is modulation of the Ca^2+^ transportome to control signaling and metabolism (Prevarskaya et al., 2018). Cell proliferation requires increased biomass and energy supply, which is provided by metabolic adaptation. Despite the presence of adequate oxygen (O_2_) levels to support mitochondrial respiration, cancer cells are highly glycolytic and, as such, demonstrate increased glucose consumption and lactate production, a phenomenon known as the Warburg effect (Vander Heiden et al., 2009). These cancer-related metabolic features are in part maintained by the activity of specific Ca^2+^ transporters, which limits mitochondrial Ca^2+^ influx and maintains glycolysis as a protective mechanism against oxidative stress. Moreover, by restricting mitochondrial Ca^2+^ entry, cancer cells reduce their vulnerability to apoptosis and cytotoxic agents (Lemeshko, 2015; Chakraborty et al., 2017). In turn, the metabolic shift toward glycolysis is a critical regulator of the Ca^2+^ supply in cancer cells. Thus, the dual pharmacological modulation of Ca^2+^ signaling and metabolism might have synergistic anticancer effects. Interestingly, preclinical evidence for the therapeutic potential of combined strategies is emerging (Park et al., 2018), yet it remains to be determined whether dual targeting will be effective in humans.

In this review, we examine the main findings regarding the interplay between Ca^2+^ and metabolism in cancer cells, with a focus on glycolysis and oxidative phosphorylation (OXPHOS). We discuss how Ca^2+^ flux between different cellular compartments affects these pivotal metabolic processes in the cell and highlight how metabolic enzymes and metabolites function as signaling molecules to influence Ca^2+^ homeostasis. We also describe how the cellular redox state defined by the metabolic features of cancer cells controls the activity of several Ca^2+^ transporters and pumps through posttranslational modifications.

The connection between Ca^2+^ and metabolism in cancer has only begun to be explored. Highlighting emerging findings on this crosstalk is important to provide further understanding of the mechanisms affecting cell function. This may yield great returns by opening up new areas of investigation and identifying new potential therapeutic opportunities.

## Calcium – Glucose Metabolism Interplay

### Ca^2+^ Regulation and Glycolysis

In all eukaryotic cells, intracellular Ca^2+^ levels are maintained at low resting concentrations (approximately 100 nM) by the activity of the major Ca^2+^ extrusion system, the plasma membrane Ca^2+^-ATPase (PMCA), which exchanges extracellular protons (H^+^) for cytosolic Ca^2+^ (Brini et al., 2013). The role played by PMCAs as housekeepers of low resting cytosolic Ca^2+^ levels is important for cell survival as it prevents Ca^2+^-dependent cell death (Brini et al., 2013). PMCAs also regulate dynamic Ca^2+^ signaling by modulating the frequency of Ca^2+^ oscillations (Caride et al., 2001).

**FIGURE 1:**
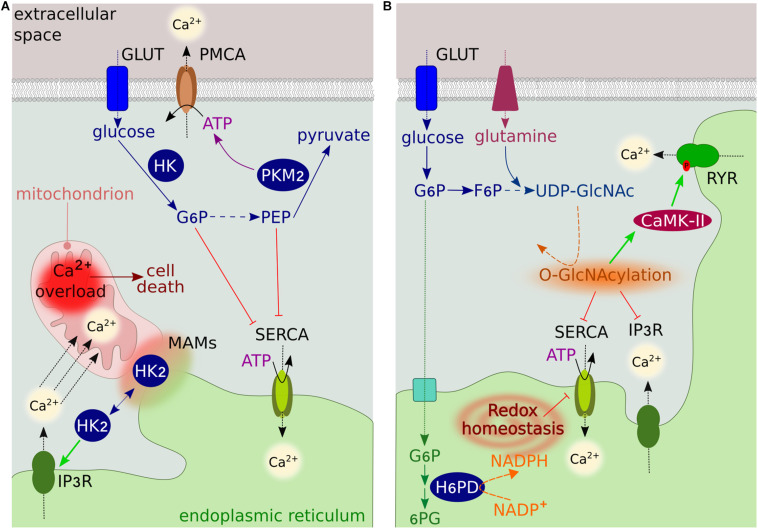
Overview of glucose metabolism-Ca^2+^ interplay. **(A)** The GLUT family transporters transfer glucose into cells, where it is converted to pyruvate though the glycolytic pathway. The rate-limiting glycolytic enzyme HK generates G6P, which inhibits ER Ca^2+^ uptake by binding SERCA. In the final step of glycolysis, PKM2 converts PEP into pyruvate, generating ATP. PEP inhibits SERCA, while ATP regulates the activity of PMCA. HK2 at MAMs modulates Ca^2+^ flux from the ER into mitochondria through IP3R to prevent mitochondrial Ca^2+^ overload-induced cell death. **(B)** The ER luminal enzyme H6PD catalyzes the first two reactions of the oxidative branch of the PPP. The conversion of G6P into 6-phosphogluconate (6PG) generates NADPH, which is used as a cofactor by several ER-reducing enzymes. By regulating the ER redox state, H6PD modulates the activity of several proteins, including SERCA and IP3R. The glycolytic intermediate fructose 6-phosphate (F6P) can enter the HBP to generate UDP-GlcNAc through a series of reactions requiring glutamine. UDP-GlcNAc modifies the activity of several Ca^2+^ transporters and regulatory proteins by O-GlcNAcylating serine and threonine residues.

It is well established that the activity of PMCAs is influenced by glycolysis, a cytosolic energy-conversion pathway that produces adenosine triphosphate (ATP) without the need for O_2_. In highly glycolytic cancer cells exhibiting the Warburg effect, the cytosolic ATP derived from glycolysis is crucial to maintaining PMCA function and cell survival (James et al., 2013, 2015). Pharmacological blockade of the glycolytic regulator 6-phosphofructo-2-kinase/fructose-2,6-bisphosphatase 3 (PFKFB3) results in PMCA inhibition, toxic cytosolic Ca^2+^ overload, and cell death (Richardson et al., 2020). Recently, a functional coupling between PMCA and the ATP-generating enzyme pyruvate kinase M2 (PKM2) has been shown in pancreatic cancer cells (James et al., 2020; Figure 1A), further supporting the notion that the cytosolic ATP supply to PMCAs may represent a potential anticancer target. Interestingly, since the activity of PMCAs is influenced by extracellular acidification, it is tempting to speculate that the highly glycolytic phenotype of cancer cells would contribute to increased Ca^2+^ efflux by providing PMCAs with an abundant source of extracellular H^+^.

In normal cells and under physiological conditions, cytosolic ATP derived from glycolysis saturates PMCA function. However, in the case of impaired mitochondrial metabolism, ATP derived from glycolysis becomes a critical regulator of PMCA activity. Acute metabolic stress inhibits the PMCA, but this effect can be attenuated by a metabolic switch from mitochondrial metabolism toward glycolysis, which restores the ATP supply to the PMCA (Mankad et al., 2012; Samad et al., 2014).

While the amount of ATP derived from glycolysis regulates PMCA activity, there is no significant evidence of regulatory feedback by the PMCA on metabolism. Indeed, silencing of PMCA4 in pancreatic ductal adenocarcinoma cells has only minimal effects on metabolic functions (Sritangos et al., 2020).

The mechanisms regulating the interplay between glycolysis and Ca^2+^ homeostasis are much more complex than simple changes in energy supply.

A first example of this complexity is related to the subcellular localization of glycolytic enzymes, which is crucial for supporting PMCA activity, and is in turn modulated by Ca^2+^. ATP is provided to the PMCA by glycolytic enzymes located in close proximity to the plasma membrane (Hardin et al., 1992; Chu et al., 2012). Studies using inside-out smooth muscle plasma membrane vesicles have demonstrated that the localized glycolytic pool of ATP regulates PMCA activity regardless of the overall cellular ATP levels (Paul et al., 1989; Hardin et al., 1993). On the other hand, cytosolic Ca^2+^ accumulation following treatment of melanoma cells with the Ca^2+^-ionophore A23187 alters the cytoskeletal compartmentalization of phosphofructokinase, thereby inhibiting glycolysis (Glass-Marmor et al., 1999). The exact mechanism is currently unknown, however, it may be related to an indirect effect of Ca^2+^ on the cytoskeletal structure.

In several cancer cells, the specific localization of the glycolytic enzyme hexokinase-2 (HK2) at mitochondria-ER contact sites [called mitochondria-associated ER membranes (MAMs)] prevents mitochondrial Ca^2+^ overload, permeability transition pore (mPTP) opening and cell death by preventing ER Ca^2+^ release through IP3R (Ciscato et al., 2020; Figure 1A). HK2 can also bind VDAC in the outer mitochondrial membrane (OMM) (Azoulay-Zohar et al., 2004; Shoshan-Barmatz et al., 2009). This association with VDAC is regulated by channel switching between an open and closed state, which is controlled by Ca^2+^ (Bathori et al., 2006). The binding reduces the channel conductance while providing HK direct access to mitochondrial ATP for enzymatic activity (Azoulay-Zohar et al., 2004), resulting in a high glycolytic rate and increased cancer cell proliferation and survival (Azoulay-Zohar et al., 2004; Abu-Hamad et al., 2008). These observations led to the hypothesis that the closure of VDAC contributes to the Warburg effect (Lemeshko, 2015).

An additional regulatory mechanism involves glycolytic metabolites. The metabolite glucose 6-phosphate (G6P) has recently been identified as a potent regulator of SERCA pumps, which transfer Ca^2+^ from the cytosol to the ER at the expense of ATP hydrolysis (Figure 1A). G6P reduces ER Ca^2+^ uptake and accumulation by directly binding SERCAs, resulting in loss of ER Ca^2+^ compartmentalization and cell death (Cole et al., 2012). This ATP-independent mechanism of regulation resembles that of the chemical SERCA inhibitor thapsigargin and is specific to G6P, as the addition of other glycolytic intermediates had no effect on ER Ca^2+^ accumulation (Cole et al., 2012).

Sarcoplasmic/endoplasmic reticulum calcium ATPase pumps are also regulated by the metabolic intermediate phosphoenolpyruvate (PEP) (Figure 1A). In cytotoxic T cells, PEP regulates the amplitude of Ca^2+^ flux and activation of the downstream nuclear factor of activated T-cell (NFAT) signaling pathway, which is critical for anti-tumor T cell functions (Ho et al., 2015). Insufficient PEP production leads to defects in Ca^2+^-NFAT signaling by increasing SERCA-mediated ER Ca^2+^ storage. Interestingly, metabolic reprogramming of T cells to generate PEP in low-glucose conditions improves anti-tumor responses (Ho et al., 2015). Although the precise molecular mechanism(s) by which this occurs remains unknown, PEP may inhibit SERCA activity by increasing its oxidative state (Ho et al., 2015).

Recently, the regulation of Ca^2+^ homeostasis by PEP has been described in two human colon carcinoma cell lines, in which increased cytosolic Ca^2+^ due to SERCA modulation stabilizes the master regulator of glycolytic metabolism, the Myc proto-oncogene protein, ultimately supporting cancer cell proliferation (Moreno-Felici et al., 2019).

While further studies are required to unravel the reciprocal regulation of Ca^2+^ levels and glycolysis in cancer cells, these examples shed light on the potential benefits of targeting those metabolic pathways that are associated with malignant transformation and regulate Ca^2+^ signaling. Most cancer cells preferentially express the M2 isoform of the pyruvate kinase enzyme catalyzing the final ATP-generating step of glycolysis in which PEP is converted to pyruvate. This provides cancer cells the advantage to adapt their metabolism depending on nutrient availability, as PKM2 switches between an active and inactive state (Dayton et al., 2016). Targeting this glycolytic enzyme may inhibit PMCA pumps specifically in cancer cells. Furthermore, the therapeutic potential of the dual inhibition of glycolysis and Ca^2+^ signaling should be considered. In support of this, combined treatment with a SERCA inhibitor (thapsigargin) and the glycolytic inhibitor 2-deoxy-d-glucose has recently shown significant anti-tumor effects in preclinical studies (Park et al., 2018).

### Contribution of Glycolytic Side Pathways

There is interesting evidence for the indirect regulation of Ca^2+^ levels by two glycolytic side branches, the pentose phosphate pathway (PPP) and the hexosamine biosynthetic pathway (HBP).

The metabolite G6P can enter the PPP, resulting in the generation of nicotinamide adenine dinucleotide phosphate (NADPH), which maintains antioxidant capacity, and ribose, the backbone of nucleotides. Recent studies in human breast cancer cells have shown that the production of NADPH by hexose-6-phosphate dehydrogenase (H6PD) in the ER lumen is indispensable for the activity of SERCAs (Figure 1B). Following ER oxidation, H6PD depletion causes an increase in SERCA expression, leading to increased ER Ca^2+^ levels and impairing cancer cell proliferation and migration (Tsachaki et al., 2018). Dysregulated SERCA expression has also been observed in *H6pd* knockout mice (Lavery et al., 2008).

These findings define, for the first time, a direct link between a metabolic pathway and ER stress, paving the way for further investigations into the ER-related metabolic pathways that sustain tumor growth by affecting Ca^2+^ homeostasis.

The activity of several proteins involved in Ca^2+^ signaling [such as calcium/calmodulin-dependent protein kinase type II (CaMK-II)], as well as Ca^2+^ pumps and channels (such as SERCA, RYR, and IP3R), is influenced by O-linked-beta-N-acetylglucosamine (O-GlcNAc) posttranslational modification (Bidasee et al., 2004; Rengifo et al., 2007; Erickson et al., 2013; Figure 1B). The amount of O-GlcNAc modification is dependent on the concentration of uridine-diphosphate-N-acetylglucosamine (UDP-GlcNAc), the end product of the HBP, which uses glucose as a substrate. Excess glucose uptake, as occurs in cancer cells, contributes to increased flux through the HBP and thus increased levels of O-GlcNAc (Jozwiak et al., 2014). The impact of this modification on channel function and activity has not been fully elucidated in cancer. Similar to other pathological conditions, increased O-GlcNAc modification is likely associated with low channel activity (Bidasee et al., 2004; Rengifo et al., 2007).

## Mitochondrial Bioenergetics and Ca^2+^ Dynamics

### Mitochondrial Membrane Potential

In vertebrates, mitochondria play an important role in ATP production and act as Ca^2+^ storage sites. The main driving force for mitochondrial Ca^2+^ uptake is the mitochondrial electrical gradient (or mitochondrial membrane potential, ΔΨm) generated by the activity of the respiratory electron transport chain (ETC) (Santo-Domingo and Demaurex, 2010). The ETC consists of four enzymatic complexes embedded in the inner mitochondrial membrane (IMM) that transport electrons delivered by reducing equivalents [nicotinamide adenine dinucleotide (NADH) and flavin adenine dinucleotide (FADH_2_)] to O_2_ while pumping H^+^ into the intermembrane space (IMS) (Marreiros et al., 2016). Together with the ΔΨm, the H^+^ gradient provides the energy to generate ATP through the F_1_F_0_ ATP synthase (or complex V) (Saraste, 1999), a process referred as OXPHOS. Furthermore, H^+^ power allows the transportation of metabolites, proteins and ions into and out of the mitochondrial matrix (Poburko and Demaurex, 2012).

Since maintenance of the ΔΨm is essential for cellular Ca^2+^ homeostasis (Contreras, 2010), it has been assumed that mitochondrial Ca^2+^ homeostasis remains normal as long as ETC dysfunction does not affect the ΔΨM. However, in recent work, Jana et al. (2019) demonstrated that mitochondrial Ca^2+^ homeostasis can be altered without changes in the ΔΨm. The authors generated two stable knockdown breast cancer cell lines for *NDUFAF3* and *SDHA* genes, encoding proteins essential for the assembly of ETC complex I and complex II, respectively. In this model, altered ETC activity was associated with reduced mitochondrial Ca^2+^ accumulation, despite no significant ΔΨm changes, suggesting that the modulation of mitochondrial Ca^2+^ may occur independently of the ΔΨm in cancer cells (Jana et al., 2019).

**FIGURE 2:**
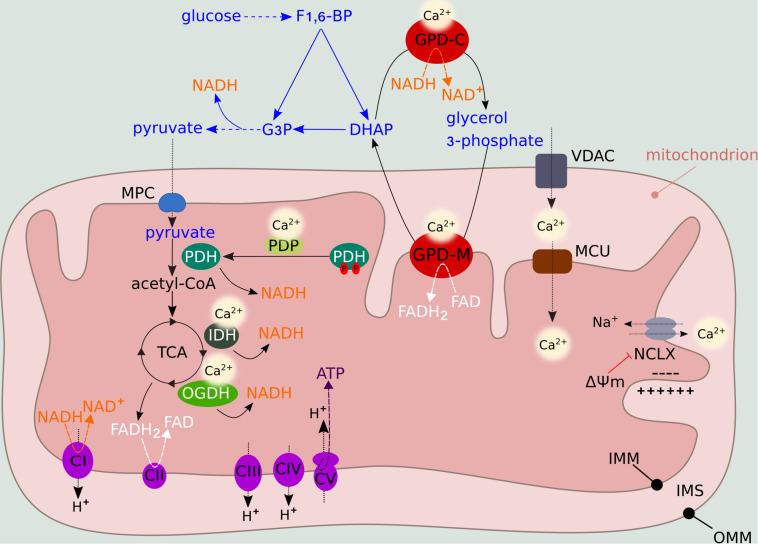
Metabolic regulation by mitochondrial Ca^2+^ levels. Mitochondrial Ca^2+^ entry through VDAC and MCU activates PDH, IDH and OGDH, providing NADH to the ETC. NADH and FADH_2_ are generated during the TCA feeding of complex I (NADH dehydrogenase) and complex II (succinate dehydrogenase), respectively. Complexes I, III, and IV (cytochrome c oxidase) function as H^+^ pumps by extruding H^+^ from the matrix to the IMS. Complex V (F_1_F_0_ ATP synthase) uses the H^+^ gradient to generate ATP in the presence of O_2_ (OXPHOS). ΔΨm fluctuations influence the activity of NCLX, the major mitochondrial Ca^2+^ efflux system. Ca^2+^ also regulates the activity of GPD-M, which constitutes the glycerol-phosphate shuttle, thus coupling mitochondrial metabolism to glycolysis. In the presence of O_2_, pyruvate derived from glycolysis enters the mitochondria via the mitochondria pyruvate carrier (MPC), where it is oxidized into acetyl-CoA. The glycolytic intermediate fructose 1,6-bisphosphate (F1,6-BP) is converted into dihydroxyacetone phosphate (DHAP) and glyceraldehyde 3-phosphate (G3P), which enters the subsequent glycolytic step, producing NADH. DHAP can be converted into glycerol-3-phosphate by GPD-C, regenerating NAD^+^ to sustain glycolysis. Subsequently, glycerol-3-phosphate is converted back into DHAP by GPD-M, converting FAD into FADH_2_.

Many metabolic pathways, including glycolysis, the tricarboxylic acid (TCA) cycle, and fatty acid oxidation, produce the electron donors that fuel the ETC. Upon exposure to high extracellular glucose concentrations, glycolysis provides a significant amount of pyruvate substrate to the TCA, elevating mitochondrial respiration and driving hyperpolarization of the IMM (Park et al., 2008). Conversely, energy deprivation of mitochondria secondary to low glucose is linked to mild mitochondrial depolarization (Gerencser et al., 2017). These glucose-driven intracellular changes control mitochondrial Ca^2+^ flux primarily via allosteric regulation of the major mitochondrial Ca^2+^ efflux system, the mitochondrial sodium/calcium exchanger protein NCLX (Kostic et al., 2018). Mild depolarization significantly reduces NCLX-mediated efflux (Figure 2). In turn, the consequent increase in matrix Ca^2+^ concentration stimulates OXPHOS, leading to repolarization that overcomes NCLX allosteric inhibition (Kostic et al., 2018). Thus, NCLX regulation by the ΔΨm links mitochondrial metabolism and Ca^2+^ signaling. This feedback mechanism can be manipulated to induce mitochondrial Ca^2+^ accumulation and cancer cell apoptosis under stress conditions such as nutrient deprivation.

### Ca^2+^ Entry in the Mitochondria and Regulation of Mitochondrial Energy Machinery

Mitochondrial Ca^2+^ uptake by the transporters VDAC (at the OMM) and MCU (at the IMM) is a key process in cell bioenergetics and functionality. In response to cellular demand, Ca^2+^ can directly modulate cellular metabolism by activating mitochondrial dehydrogenase enzymes and regulating ETC function (Denton, 2009).

By controlling the activity of Ca^2+^-sensitive pyruvate dehydrogenase phosphatase (PDP), Ca^2+^ regulates the dephosphorylation state, and hence activation, of pyruvate dehydrogenase (PDH) complex, thus feeding the TCA (Figure 2). Two TCA enzymes, NAD-isocitrate dehydrogenase (IDH) and mitochondrial 2-oxoglutarate dehydrogenase (OGDH, also known as alpha-ketoglutarate dehydrogenase), which generate NADH for ATP synthesis, are allosterically activated by mitochondrial Ca^2+^ levels (Figure 2). The fourth enzyme, mitochondrial glycerol-3-phosphate dehydrogenase (GPD-M), constitutes the glycerol-phosphate shuttle and transfers reducing equivalents from NADH (produced by glycolysis) to the ETC. As for IDH and OGDH, the effect of Ca^2+^ on GPD-M is to lower the dissociation constant of the substrate (Denton, 2009; Figure 2).

The stimulation of TCA dehydrogenases by Ca^2+^ triggers the activation of mitochondrial metabolic machinery, leading to increased ATP production to balance the energy demands of cancer cells during periods of increased proliferation. Inhibition of mitochondrial Ca^2+^ influx by genetic manipulation of the MCU complex in HeLa cells disrupts OXPHOS, lowers cellular ATP levels and activates 5′-adenosine monophosphate (AMP)-activated protein kinase catalytic subunit alpha-2 (AMPK)-dependent pro-survival autophagy (Mallilankaraman et al., 2012). A similar phenotype has been described following inhibition of IP3R-mediated ER Ca^2+^ release (Cardenas et al., 2010), suggesting that Ca^2+^ transfer from the ER to mitochondria is crucial for optimal cellular bioenergetics. Conversely, deprivation of mitochondrial TCA substrates alters mitochondrial Ca^2+^ flux by regulating the expression of the gatekeeper of the mitochondrial calcium uniporter MICU1, thereby protecting the cells from Ca^2+^ overload under conditions of limiting metabolite availability (Nemani et al., 2020). Many cancer cells are thought to have defective mitochondria, thus relying primarily on aerobic glycolysis. However, interestingly, a recent paper has shown that Ca^2+^ signaling in mitochondria remains a critical regulator of mitochondrial metabolism even in cells with impaired OXPHOS. In these cells, mitochondrial Ca^2+^ uptake is crucial to sustain OGDH-mediated metabolic reprogramming, and thus cancer cell survival (Cardenas et al., 2020). This evidence suggests that dual targeting of metabolism and Ca^2+^ shuttling to mitochondria may have synergistic effects in specific cancer subtypes.

Several other studies have shown that the close proximity of mitochondria to Ca^2+^ transporters in the ER is fundamental to determining mitochondrial Ca^2+^ handling properties (Rutter and Rizzuto, 2000). Increased ER-mitochondria coupling leads to cancer cell death through mitochondrial Ca^2+^ overload, while a reduction in contact sites shifts bioenergetics to glycolysis and contributes to therapy resistance (Cardenas et al., 2016; Madreiter-Sokolowski et al., 2016).

The direct link between mitochondrial Ca^2+^ levels and mitochondrial ATP production has been established by the seminal work of the Rizzuto group (Jouaville et al., 1999). By using mitochondrial aequorin as a targeted recombinant Ca^2+^ probe and an ATP-sensitive luciferase, the authors showed that stimulation of HeLa cells with histamine, which releases Ca^2+^ from ER stores, induces an increase in both mitochondrial Ca^2+^ and ATP levels. Interestingly, in the presence of pyruvate and lactate (oxidative substrates), ATP synthesis continued for several minutes after the mitochondrial Ca^2+^ signal returned to basal levels, suggesting the existence of a metabolic memory imposed by Ca^2+^ in cancer cells (Jouaville et al., 1999). This mechanism is currently unknown; however, it may involve persistent Ca^2+^ activation of mitochondrial dehydrogenases (Robb-Gaspers et al., 1998), prolonged changes in the ΔΨm (Jouaville et al., 1999) or activation of ATP synthase (see below). An alternative possibility is that of Ca^2+^-induced long-lasting events, such as activation of the ETC by posttranslational modifications (Glancy et al., 2013) or changes in mitochondrial volume (Halestrap, 1989).

Nevertheless, while Ca^2+^-mediated activation of dehydrogenases in mitochondria is well established, recent reports have questioned the importance of mitochondrial Ca^2+^ levels in controlling substrate supply for OXPHOS. Surprisingly, mitochondrial OXPHOS is not altered in MCU knockout mice, suggesting that additional mechanisms are involved in OXPHOS regulation (Szibor et al., 2020). Experimental findings have demonstrated that the malate/aspartate shuttle (MAS) acts as the “gas pedal” for mitochondrial metabolism by providing a supply of cytosolic pyruvate for OXPHOS (Szibor et al., 2020). An essential component of the MAS is the Ca^2+^-binding mitochondrial carrier protein Aralar1/2, an aspartate/glutamate transporter, which exchanges glutamate and one H^+^ (from the cytosol) for one aspartate (from the mitochondria) (Palmieri et al., 2001). Since the Ca^2+^-regulatory binding site of Aralar1/2 faces the mitochondrial IMS, MAS senses cytosolic, but not mitochondrial, Ca^2+^ levels (Palmieri et al., 2001). Thus, cytosolic Ca^2+^ controls the pyruvate supply to the mitochondria, while mitochondrial Ca^2+^ accounts for only 15% of the pyruvate-driven OXPHOS rate (Szibor et al., 2020). While mathematical models and additional experimental findings corroborate these observations, the debate is still ongoing (Gellerich et al., 2012; Korzeniewski, 2017), as findings are further complicated by compensatory metabolic mechanisms, as well as cell-type and tissue-specific effects (Pan et al., 2013; Boyman et al., 2020).

In addition to Aralar1/2, ATP-magnesium/phosphate carriers are regulated by Ca^2+^. This shuttle system catalyzes the exchange of ATP-magnesium ions (Mg^2+^) and adenosine diphosphate (ADP)-Mg^2+^ for one phosphate across the IMM, thus determining the levels of adenine nucleotides (AMP, ADP, ATP) in the mitochondria. The N-terminal region of these carriers contains Ca^2+^-binding domains that respond to high Ca^2+^ levels (Nosek et al., 1990). An unbalanced exchange capacity of these carriers can alter the concentration of the adenine nucleotide pool in the matrix, thus impacting cellular growth and energy metabolism.

Recently, it has been proposed that Ca^2+^ can directly modulate the enzymatic activity and velocity of F_1_F_0_ ATP synthase (Territo et al., 2000). Indeed, pharmacological blockade of mitochondrial Ca^2+^ entry leads to reduced ATP synthase activity (Das and Harris, 1990). However, the underlying mechanism is unknown, and evidence for Ca^2+^ binding or direct regulation of the ETC is still limited (Hubbard and McHugh, 1996; Wescott et al., 2019).

In general, as discussed above, increased mitochondrial Ca^2+^ levels result in increased mitochondrial metabolism. However, this is not always the case. When Ca^2+^ levels increase to just below the threshold sufficient to cause mPTP opening, the ability of mitochondria to synthesize ATP is compromised (Pandya et al., 2013). Most of the evidence to date has attributed this effect to inhibition of mitochondrial dehydrogenases, loss of purine nucleotides, and reduced ΔΨm (Fagian et al., 1986; Lai et al., 1988; Fink et al., 2017). Moreover, a recent study suggested that inhibition of ETC complex I is the most likely cause of the decrease in OXPHOS through accumulation of mitochondrial Ca^2+^ as phosphate precipitates (Malyala et al., 2019).

Ca^2+^ signals can also influence mitochondrial bioenergetics independent of ATP production. In response to repetitive cytosolic Ca^2+^ oscillations, NADH generated in the mitochondria is not used as a substrate for ATP synthesis, but it is transported (via MAS) to the cytosol. Once located in the cytosol, NADH influences the activity and expression of sirtuins (Marcu et al., 2014). This family of proteins comprises NAD-dependent deacetylases, thus regulating the posttranslational modifications of multiple targets, including metabolic genes (Zhao et al., 2010). Thus, mitochondrial Ca^2+^ accumulation impacts the regulation of metabolic adaptation by influencing protein acetylation and epigenetics.

Recent studies have provided evidence for this link. Alteration of mitochondrial metabolism upon VDAC1 depletion in glioblastoma cells limits the production of essential metabolic mediators of epigenetic processes, such as NADH, citrate, and acetyl-CoA (Arif et al., 2017; Amsalem et al., 2020). The associated changes in histone acetylation and methylation have profound effects on tumor growth, suggesting that this intricate link between Ca^2+^, metabolism and epigenetics may be exploited as an innovative therapeutic strategy for glioblastoma and other cancers (Arif et al., 2017; Amsalem et al., 2020).

## Redox State and Ca^2+^ Balance

### Oxidative Stress as a Modulator of Ca^2+^ Signaling

Mitochondria produce ROS as a natural byproduct of the activity of ETC. Due to electron leakage during respiration, O_2_ is reduced to form the radical superoxide anion (O_2_^–^), which is rapidly converted into non-radical hydrogen peroxide (H_2_O_2_), the major source of oxidative stress in cells (Schieber and Chandel, 2014). An increased metabolic rate results in more O_2_ consumption, resulting in increased ETC leakage and higher ROS levels (Zhao et al., 2019).

The interplay between Ca^2+^ and ROS is well established, with Ca^2+^ signaling influencing the cellular generation of ROS and oxidants regulating the activity of many Ca^2+^ channels and transporters.

**FIGURE 3:**
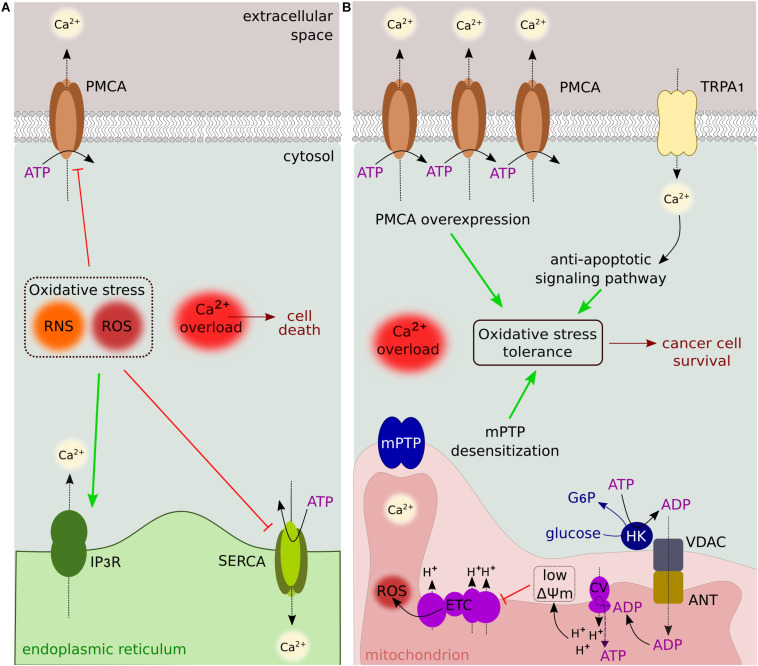
Oxidative stress and Ca^2+^ signaling. **(A)** Reactive nitrogen/oxygen species alter the sensitivity of transporters and pumps to Ca^2+^. Cytosolic Ca^2+^ overload is induced by IP3R stimulation, and SERCA/PMCA inhibition. **(B)** Oxidative stress tolerance is sustained by several Ca^2+^-dependent mechanisms, including PMCA overexpression, activation of anti-apoptotic signaling pathways, and mPTP desensitization. The binding of HK to VDAC contributes to reduced O_2_^–^ leakage by lowering ΔΨm. These mechanisms are used by cancer cells to mediate resistance to ROS-producing anticancer drugs.

Changes in the cellular redox state induce cytosolic Ca^2+^ overload by stimulating Ca^2+^ release from the ER (via IP3R), decreasing SERCA activity and inhibiting Ca^2+^ extrusion from the plasma membrane (Camello-Almaraz et al., 2006; Qin et al., 2013; Figure 3A). These events are associated with redox modifications that alter the sensitivity of the transporters to Ca^2+^ (Zaidi et al., 2003; Sharov et al., 2006; Joseph et al., 2018). The increase in cytosolic Ca^2+^ results in transient mPTP opening (called “flicker”) to prevent cell death by Ca^2+^ overload. mPTP Ca^2+^ desensitization and PMCA overexpression are escape mechanisms used by cancer cells to increase their resistance to death (Rasola and Bernardi, 2015; Peters et al., 2016). Recently, the redox-sensing transient receptor potential cation channel (TRP) subfamily A member 1 (TRPA1) has been shown to upregulate Ca^2+^-dependent anti-apoptotic signaling pathways in response to oxidative stress, allowing cancer cells to adapt to therapy by promoting oxidative stress tolerance (Takahashi et al., 2018; Figure 3B).

Mitochondrial ROS accumulation further augments ROS production through a positive feedback mechanism, overall increasing mitochondrial Ca^2+^ uptake by altering VDAC and MCU activity via redox modifications (De Pinto et al., 2016; Dong et al., 2017).

Another major endogenous source of ROS in cells is the NADPH oxidase isoform family (NOX1-5); these enzymes generate O_2_^–^ and H_2_O_2_ in specific cell compartments, including the ER and plasma membrane (Ushio-Fukai, 2006). Although the mechanisms of NOX regulation of Ca^2+^ ion channels are not completely understood, the majority of NOX-derived ROS have been shown to induce extracellular Ca^2+^ entry. In cancer cells, modulation of NOX expression to manipulate ROS-dependent intracellular Ca^2+^ signaling could potentially be used to inhibit cancer progression (Song et al., 2014). However, current evidence is still mixed, as redox regulation can have different consequences depending on the specific NOX isoform involved and the cell context. For example, the interplay between NOX3 and transient receptor potential cation channel subfamily V member 1 (TRPV1) appears to be implicated in chemotherapy-induced toxicity through the augmentation of oxidative stress and regulation of cell death (Mukherjea et al., 2008).

In addition to channels, several other proteins involved in Ca^2+^ signaling are targeted by ROS-dependent oxidation. Oxidants generated by mitochondria can oxidize, among others, CaMK-II, thus enhancing its activity (Erickson et al., 2008). Following activation, CaMK-II regulates numerous Ca^2+^-regulatory proteins, including SERCA and RYR, leading to aberrant intracellular Ca^2+^ handling (Hook and Means, 2001). Considering the involvement of CaMK in several Ca^2+^-dependent cell processes, such as proliferation, apoptosis, and migration (Wayman et al., 2011), it is not surprising that its activity has been found to be aberrant in many cancer types (Brzozowski and Skelding, 2019).

Cells with high metabolic demand, such as cancer cells, also generate reactive nitrogen species (RNS). RNS are formed by peroxidase- and nitric oxide synthase (NOS)-mediated enzymatic reactions, as well as via the interaction of nitric oxide (NO) with ROS (Grisham et al., 1999).

Although very little is known about the regulation of Ca^2+^ responses by RNS, most of the effects are attributed to posttranslational modifications that generate nitrosylation changes in cysteine residues of Ca^2+^ transporters, channels and pumps, altering their activity (Xu et al., 1998; Yoshida et al., 2006; Zhou et al., 2015). In addition, NO derivatives can induce irreversible oxidation (Adachi et al., 2004; Tong et al., 2008). These changes are functionally associated with the inhibition of Ca^2+^ pumps and activation of Ca^2+^ release and entry channels, thus increasing the overall cytoplasmic Ca^2+^ level. Experimental evidence in cancer cells suggests that RNS are involved in mediating anti-tumor effects through Ca^2+^-dependent cell death (Tokunaga et al., 2018); however, further studies are needed to elucidate the mechanisms of RNS-regulated Ca^2+^ dynamics.

### Ca^2+^ Homeostasis and ROS Generation

In cancer cells, elevated ROS levels below the threshold causing cell damage stimulate tumorigenesis by activating signaling pathways that regulate cancer cell proliferation, angiogenesis and metabolic reprogramming. In contrast, excessive ROS leads to cell injury and death (Sullivan and Chandel, 2014). Thus, ROS levels must be kept under tight control by balancing ROS generation and scavenging.

Changes in Ca^2+^ levels may impact cancer progression by affecting protumorigenic ROS levels and inducing apoptosis. As a consequence of supporting mitochondrial metabolism, mitochondrial Ca^2+^ can impact ETC-dependent ROS production and redox signaling by directly stimulating mitochondrial ROS-generating enzymes, such as GPD-M and OGDH. Moreover, ER-mitochondrial Ca^2+^ transfer stimulates ROS mobilization from the mitochondria, leading to the formation of redox nanodomains at MAMs, which in turn modulate cytosolic Ca^2+^ oscillations (Booth et al., 2016).

Excessive oxidative stress by mitochondrial Ca^2+^ accumulation induces apoptosis (Wang et al., 2015), while moderate ROS levels following inactivation of MICU1 support tumor growth by sustaining protumoral signaling pathways (Marchi et al., 2019). Similarly, expression of the MCU regulator mitochondrial calcium uniporter regulator 1 (MCUR1) stimulates ROS-mediated cancer cell migration and survival, accelerating tumor growth (Ren et al., 2018; Jin et al., 2019; Liu et al., 2020). Targeting MCU-mediated ROS production reduces the invasive properties of cancer cells and suppresses tumor growth and metastasis *in vivo* (Tosatto et al., 2016; Ren et al., 2017). However, these effects are likely tumor-specific, as other studies have reported conflicting evidence. Depending on experimental conditions, no changes in ROS levels have been observed upon alteration of mitochondrial Ca^2+^ uptake (Hall et al., 2014). Moreover, Ca^2+^ can counterintuitively enhance ROS production when complex I is inhibited (Sousa et al., 2003).

In addition to MCU-mediated effects on ROS output, Ca^2+^ entry via transient receptor potential channels (TRPs) plays an important role in modulating mitochondrial ROS levels. Upon inhibition of TRP cation channel melastatin subfamily member 2 (TRPM2), decreased intracellular Ca^2+^ levels result in reduced expression of mitochondrial genes and mitochondrial dysfunction (Bao et al., 2016). High ROS levels in neuroblastoma cells expressing a dominant-negative form of TRPM2 are associated with increased susceptibility to chemotherapeutic drugs and reduced cell survival and tumor growth (Bao et al., 2016).

The binding of the glycolytic HK enzyme to VDAC has a protective antioxidant role. ADP generated by HK during the conversion of glucose to G6P is used in an ADP/ATP recycling process involving the direct interaction between VDAC and the adenine nucleotide translocator (ANT), keeping ADP at steady-state levels. This in turn sets the ΔΨm to lower levels and decreases O_2_^–^ leakage (Figure 3B). When this cycling is affected, ROS production is increased and associated with oxidative stress (da-Silva et al., 2004). Thus, VDAC-HK association not only provides a metabolic advantage (see above) but also plays a major role in regulating apoptosis via redox signaling.

The regulation of the redox balance by VDAC also involves mitochondrial ROS efflux to the cytosol, as VDAC acts as a release channel for hydrophilic O_2_^–^ (Han et al., 2003). Indeed, VDAC closure increases mitochondrial oxidative stress, sensitizing cells to Ca^2+^-induced mPTP opening and apoptosis (Tikunov et al., 2010). These findings have been corroborated by a recent study showing a positive correlation between VDAC levels and the expression of two subunits of ETC complex I, the major source of mitochondrial ROS (Mota-Martorell et al., 2020).

Finally, a yet unexplored possibility is that Ca^2+^ contributes to ROS production by regulating cellular antioxidant defenses. For example, increased intracellular Ca^2+^ concentrations may generate more ROS because of impaired levels of reduced glutathione (GSH), the most important antioxidant synthesized in cells. Interestingly, the existence of a Ca^2+^-sensitive GSH transporter in the ER has been recently postulated (Lizak et al., 2020), and its activity might be differentially regulated in cancer.

## Conclusion

It has become increasingly evident that Ca^2+^ impacts multiple metabolic pathways within cells and that several Ca^2+^ channels are much more than simple ion transporters. An example is the activity of mitochondrial transporters VDAC and MCU, which have been found to be responsible for aberrant metabolism that supports cancer progression and therapy resistance (Azoulay-Zohar et al., 2004; Abu-Hamad et al., 2008; Chakraborty et al., 2017).

The hypothesis that cancer cells have dysfunctional mitochondrial metabolism and that the increase in glycolytic rate is a compensatory mechanism (Warburg’s theory) has been called into question by evidence that mitochondrial metabolism is essential for cancer biology. Increased mitochondrial Ca^2+^ uptake stimulates mitochondrial metabolism, which provides the building blocks and energy to sustain uncontrolled proliferation. Moreover, mitochondrial metabolism produces ROS, which activate signaling pathways supporting tumorigenesis, such as hypoxia and inflammation pathways (Tosatto et al., 2016; Liu et al., 2020).

An interesting observation is the mitochondrial Ca^2+^ addiction of cancer cells with defective oxidative metabolism. In these cells, Ca^2+^ transfer to mitochondria sustains survival by mediating the compensatory metabolic switch toward reductive carboxylation. Thus, specific anti-metabolic drugs may be effective as combination therapy with drugs that target mitochondrial Ca^2+^ transfer, regardless the OXPHOS status of cancer cells.

The relationship between glycolysis and Ca^2+^ is complex. In addition to the ATP-dependent regulation of Ca^2+^ pumps, the physical association of glycolytic intermediates with Ca^2+^ transporters modulates Ca^2+^ homeostasis, while changes in the subcellular localization of glycolytic enzymes influence cell fate by impacting Ca^2+^ homeostasis (Ciscato et al., 2020). Future investigations should focus on the cellular compartmentalization of metabolic intermediates as demonstrated for Ca^2+^. It is becoming highly feasible to analyze regional changes in metabolites and metabolic activities in a topographic dimension. The next step is to combine these imaging tools with those used to study Ca^2+^ compartmentalization.

Clearly, many gaps remain in terms of our understanding of Ca^2+^-metabolism communication. To this point, a key aspect that we should consider is experimental design. Early reports used Ca^2+^ concentrations exceeding normal physiological levels, providing evidence of regulatory mechanisms that may not be highly relevant to the normal physiological conditions that precede pathological transformation. Moreover, it is quite unlikely that findings obtained in (isolated) cells in culture would be valuable in organ systems as well.

One of the greatest challenges is restoring physiological Ca^2+^ levels or manipulating Ca^2+^ dynamics to selectively kill cancer cells. Unfortunately, the limited number of specific inhibitors has prevented the development of efficient anticancer strategies targeting aberrant Ca^2+^ signaling. An alternative strategy is to target the dysfunctional metabolic pathways that also regulate Ca^2+^ signaling, such as glycolysis. For example, targeting the glycolytic enzymes PFKFB3 and PKM2 which are highly expressed in cancer cells compared to normal cells, would provide a novel means of targeting Ca^2+^ pumps specifically in cancer cells. Given this possibility and the therapeutic potential of a synergistic approach to, for instance, reduce tumor acidification or induce Ca^2+^-dependent apoptosis in cancer cells during glucose deprivation, it is crucial to further dissect how Ca^2+^ levels and metabolism integrate to regulate tumorigenesis and cancer progression.

Finally, the possibility of targeting metabolic pathways and Ca^2+^ dynamics to manipulate the epigenome is a fascinating idea. New findings are emerging (Arif et al., 2017; Lombardi et al., 2019; Amsalem et al., 2020), and it will now be critical to understand the mechanisms by which concerted alterations in metabolism and Ca^2+^ signaling regulate epigenetic-mediated transcriptional control in cancer cells.

## Author Contributions

ARC conceived the review and wrote, reviewed, and edited the text. CD provided a first draft of the manuscript and designed the figures. DG reviewed the text. All authors performed literature searching and contributed to discussion of the content.

## Conflict of Interest

The authors declare that the research was conducted in the absence of any commercial or financial relationships that could be construed as a potential conflict of interest.

## Abbreviations

6PG: 6-phosphogluconate
ADP: adenosine diphosphate
AMP: 5 ′ -adenosine monophosphate
AMPK: 5 ′ -AMP-activated protein kinase catalytic subunit alpha-2
ANT: adenine nucleotide translocator
ATP: adenosine triphosphate
Ca^2+^: calcium ions
CaMK-II: calcium/calmodulin-dependent protein kinase type II
DHAP: dihydroxyacetone phosphate
ER: endoplasmic reticulum
ETC: electron transport chain
F1,6-BP: fructose 1,6-bisphosphate
F6P: fructose 6-phosphate
FADH2: flavin adenine dinucleotide
G3P: glyceraldehyde 3-phosphate
G6P: glucose 6-phosphate
GPD-C: cytoplasmic glycerol-3-phosphate dehydrogenase
GPD-M: mitochondrial glycerol-3-phosphate dehydrogenase
GSH: reduced glutathione
H^+^: proton
H_2_O_2_: hydrogen peroxide
H6PD: hexose-6-phosphate dehydrogenase
HBP: hexosamine biosynthetic pathway
HK(s/1/2): hexokinase(s)-1/2
IDH: NAD-isocitrate dehydrogenase
IMM: inner mitochondrial membrane
IMS: intermembrane space
IP3: inositol 1,4,5-triphosphate
IP3R: inositol 1,4,5-triphosphate receptor
MAMs: mitochondria-associated endoplasmic reticulum membranes
MAS: malate/aspartate shuttle
MCU: mitochondrial calcium uniporter protein
MCUR1: mitochondrial calcium uniporter regulator 1
Mg^2+^: magnesium ions
MICU(1): mitochondrial calcium uptake protein (1)
MPC: mitochondria pyruvate carrier
mPTP: mitochondrial permeability transition pore
NAD(H): nicotinamide adenine dinucleotide
NADP(H): nicotinamide adenine dinucleotide phosphate
NCLX: mitochondrial sodium/calcium exchanger protein
NFAT: nuclear factor of activated T-cells
NO: nitric oxide
NOS: nitric oxide synthase
NOX(s/1-5): NADPH oxidase(s) (1-5)
O-GlcNAc: O-linked -beta-N-acetylglucosamine
O_2_: oxygen
O_2_^–^: oxygen superoxide anion
OGDH: mitochondrial 2-oxoglutarate dehydrogenase
OMM: outer mitochondrial membrane
OXPHOS: oxidative phosphorylation
PDH: pyruvate dehydrogenase
PDP: pyruvate dehydrogenase phosphatase
PEP: phosphoenolpyruvate
PFKFB3: phosphofructokinase fructose bisphosphatase-3
PKM2: pyruvate kinase M2
PMCAs: plasma membrane Ca^2+^-ATPases
PPP: pentose phosphate pathway
RNS: reactive nitrogen species
ROS: reactive oxygen species
RYR: ryanodine receptor
SERCAs: sarcoplasmic/endoplasmic reticulum calcium ATPases
TCA: tricarboxylic acid cycle
TRPA1: transient receptor potential cation channel subfamily A member 1
TRPM2: Transient receptor potential cation channel subfamily M member 2
TRPs: transient receptor potential channels
TRPV1: transient receptor potential cation channel subfamily V member 1
UDP-GlcNAc: uridine-diphosphate-N-acetylglucosamine
VDAC(1): voltage-dependent anion-selective channel protein (1)
ΔΨ m: mitochondrial membrane potential.
